# Transvaginal closure of urinary bladder opening and Mitrofanoff technique in a neurologically impaired female with chronic indwelling catheter: a case presentation

**DOI:** 10.1186/s12894-021-00861-0

**Published:** 2021-06-27

**Authors:** Athanasios Zachariou, Minas Paschopoulos, Aris Kaltsas, Fotios Dimitriadis, Athanasios Zikopoulos, Charalampos Mamoulakis, Atsushi Takenaka, Nikolaos Sofikitis

**Affiliations:** 1 Urology Department, Medical School, University of Ioannina, Ioannina, Greece; 2 Department of Obstetrics and Gynaecology, Medical School, University of Ioannina, Ioannina, Greece; 3 Urology Department, Medical School, Aristotle University of Thessaloniki, Thessaloniki, Greece; 4 Urology Department, Medical School, University of Crete, Heraklion, Greece; 5 Urology Department, Medical School, Tottori University, Yonago, Japan; 6 3 Spyridi Street, 38221 14 Volos, Greece

**Keywords:** Urethral damage, Indwelling catheter, Transvaginal urethral closure, Mitrofanoff, Case report

## Abstract

**Background:**

Chronic catheterization remains the only attractive option in specific circumstances, especially in neurologically impaired patients. Complications produced by the indwelling catheters, like patulous urethra and bladder neck destruction, usually lead to severe incontinence and significant nursing difficulties. Here, we describe a rare case, a urinary bladder opening representing massive and extensive destruction of the urethra and bladder sphincter due to an indwelling catheter.

**Case presentation:**

We present a 46-year-old paraplegic woman complaining of recurrent febrile urinary tract infections and severe urinary incontinence. She suffered from persistent malodorous urine and skin breakdowns from constant urine leakage. The vaginal examination revealed extensive destruction of the urethra and a 10 cm opening permitting the urinary bladder wall to prolapse into the vagina. The patient underwent a combined surgical approach; a transvaginal bladder closure with anterior colporrhaphy and a Mitrofanoff procedure to ensure a continent stoma for future clean intermittent self-catheterization (CISC). The patient is compliant with CISC and, remains continent twelve years after surgery.

**Conclusion:**

This case demonstrates that in the era of CISC, there are still neurologically impaired females suffering from rare but critical adverse effects of indwelling catheters. The urethra and bladder neck erosion represent a demanding treatment assignment. The Mitrofanoff procedure for continent stoma and the transvaginal closure of urinary bladder opening produced a lifesaving potential treatment.

## Background

Clean intermittent self-catheterization (CISC) has become generally accepted during the last decades as the gold standard for the management of neurogenic bladder. The principles of CISC are to empty the bladder efficiently, to avoid complications and to improve storage function [1]. The main reason for the institution of an indwelling catheter is the challenge in controlling urinary incontinence in female patients. Chronic catheterization remains an attractive option only in specific conditions. These are the impaired upper limb function in high cervical spine injury patients, the absence of a caregiver to perform clean intermittent catheterization and the lack of satisfactory external collection device in the females [2].

There are several reports in the literature describing the complications of long term indwelling catheterization. These include bladder and peritoneal perforation [3], urinary infections [4], bladder calculi and catheter encrustations [5], catheter complications like blockage [6], urethral stenosis, urethral damage and malignancy associated with chronic infection [7]. In a multicenter cohort study of 2076 adults with an indwelling urethral catheter, 57% of patients reported at least 1 complication because of the catheter, and noninfectious complications (55%) were 5 times as common as infectious complications (11%) [8].

One of the most challenging problems in the management of women with neurogenic bladders is incontinence around their indwelling catheter. Anticholinergics, β_3_-adrenoceptor agonist and a larger catheter, resolve urinary leakage for a limited time. The use of larger caliber catheters, can produce patients facing acute adverse effects [9]. For example, pressure necrosis from long-term indwelling catheters and extrusion of a Foley catheter during sudden movements damage the urethra. Complications produced by the catheters, like patulous urethra and bladder neck destruction, usually lead to significant nursing difficulties. Severe incontinence increases the depression of these patients, who are often bedridden or wheelchair-bound and may also be suffering from coexisting pressure sores.

In the present study, a rare case is described; a urinary bladder opening representing massive and extensive destruction of the urethra and bladder sphincter due to indwelling catheter in a neurologically impaired woman. The combined use of two surgical approaches, the transvaginal closure of bladder opening and the Mitrofanoff technique, produced a urinary bladder with a continent stoma. Furthermore, the reason to publish our case now was that there is a long term evaluation of the proposed therapy since more than a decade has passed since the procedures. Finally, emphasis should be given to the fact that in the era of CISC, there are still unusual critical complications concerning indwelling catheters and a combination of rehabilitation surgical techniques are always crucial for radical treatment.

## Case presentation

A 46-year-old paraplegic woman presented to the outpatient urology department, complaining of recurrent febrile urinary tract infections and severe urinary incontinence for 2 years. She suffered from persistent malodorous urine and skin breakdowns from constant urine leakage. She typically used long-term urethral catheters, which had resulted in dilatation and pressure necrosis of the urethra with subsequent severe incontinence.

**Fig. 1:**
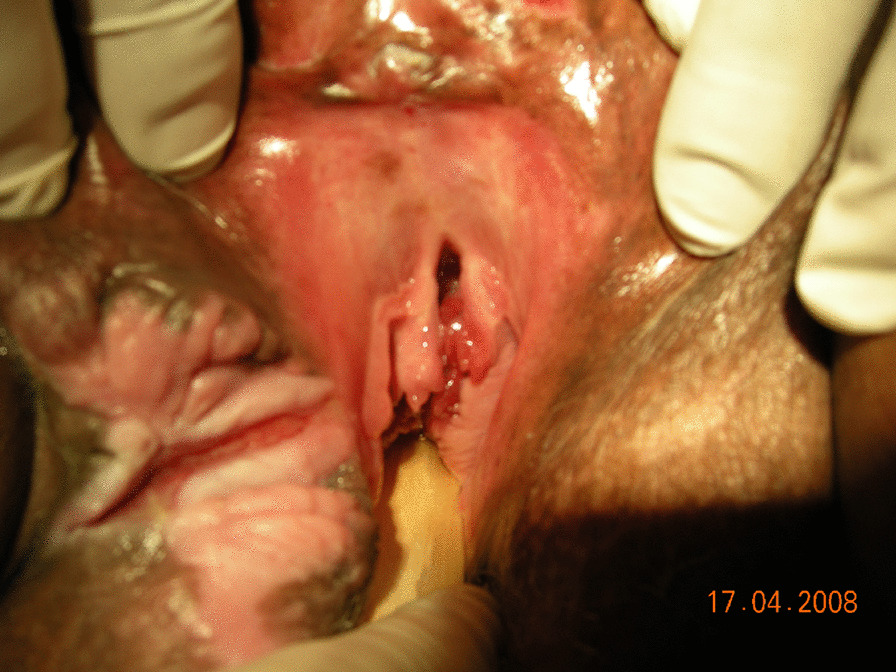
The vaginal examination revealed destruction of the urethra

**Fig. 2:**
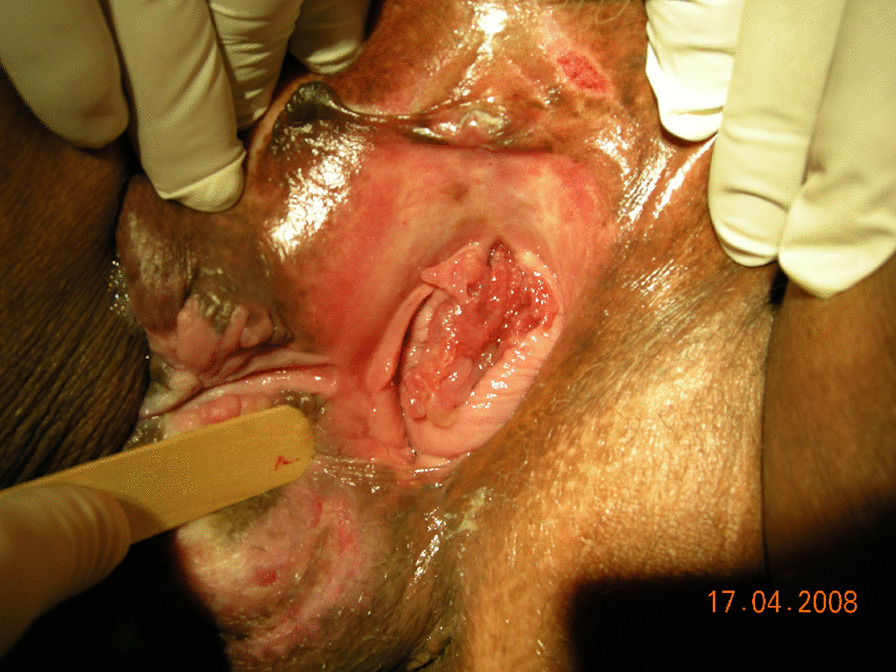
The vaginal examination revealed a 10 cm opening permitting the urinary bladder wall to prolapse into the vagina

The vaginal examination revealed destruction of the urethra and a 10 cm opening permitting the urinary bladder wall to prolapse into the vagina (Figs. 1, 2). Three fingers could be easily inserted into the urethra, and the bladder wall could practically be observed from the outside. A diffuse scar tissue area had replaced bladder trigone, and ureteral orifices were visible through the bladder opening. An intravenous urography was performed to check the anatomy of the upper urinary tract, which was within normal limits. The computed tomography (CT) scan did not assist in the diagnosis because of the artifacts caused by the bilateral total hip replacements.

The patient underwent a combined transvaginal urethral closure with anterior colporrhaphy and a Mitrofanoff procedure to ensure a continent stoma for future CISC. The patient was placed initially in the dorsal lithotomy position, and a retractor was placed in the vagina. The labia were retracted laterally with stay sutures. The damaged bladder wall was circumcised, and an anterior vaginal flap was raised. Special care was taken to entirely free the lateral part of the bladder base and the attachments of the pubic bone. There was extensive destruction of pubourethral ligaments, and that allowed a complete mobilization of the bladder wall from the symphysis pubis. The opening was closed with a 2/0 polyglactin (Vicryl™) suture. A second layer, using the same suture, was applied to invaginate further the bladder opening to ensure an excellent postoperative result. An anterior colporrhaphy was accomplished to push anteriorly further the bladder (third layer). Finally, the anterior vaginal flap was advanced to cover the area where the previous urethra and bladder neck were present, forming the last (fourth) layer of closure.

A laparotomy followed immediately after that, and an appendicectomy was done, maintaining its blood supply. The tip of the appendix was transected, and a catheter was introduced to ensure patency. A Mitrofanoff procedure was performed, and the appendix brought out through umbilicus. A small cystostomy was made, and the bladder mucosa was prepared to accomplish an appendico-vesical anastomosis. Recovery from this procedure took six weeks. Initially, the Mitrofanoff channel was not used for draining urine. During this time, a suprapubic catheter was inserted to drain urine. The suprapubic catheter was removed once CISC through the Mitrofanoff channel started. The suprapubic catheter opening closed easily.

**Fig. 3:**
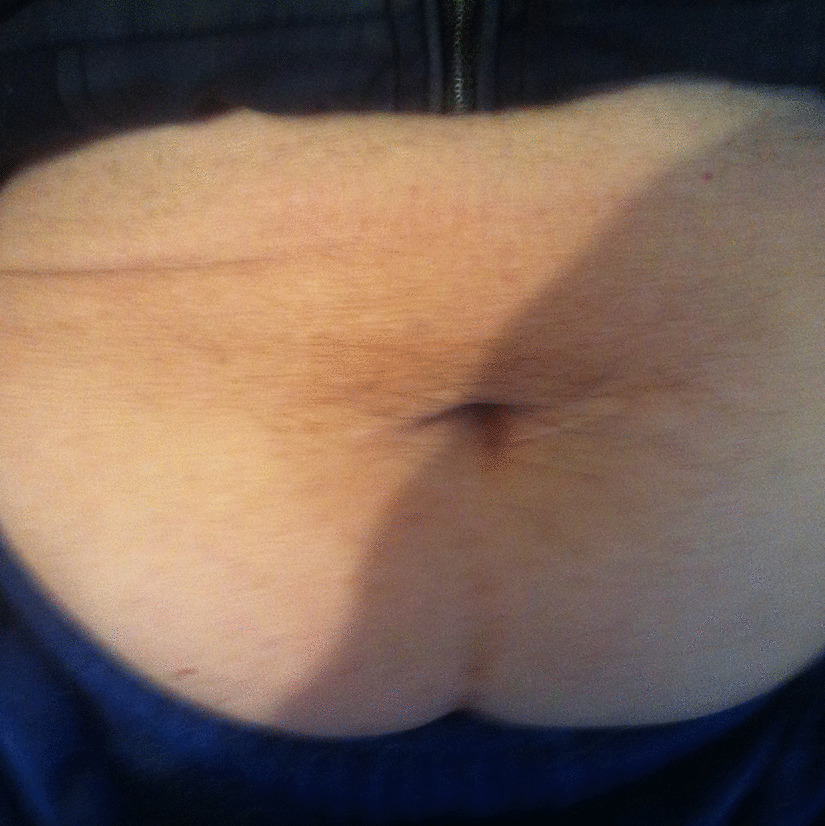
The Mitrofanoff continent stoma

During the initial postoperative period, the patient received oxybutynin for three months. The patient is compliant with CISC and remains continent twelve years after surgery. She has a normal renal function with no upper tract dilatation on ultrasonography. The woman underwent evaluation recently, and she is still dry with no urinary fistula reported (Fig. 3).

The patient reported complications concerning recurrent urinary tract infections and urine leakage when delays the emptying. She performs four CISC per day and undergoes annual evaluation with an ultrasound examination to ensure that any stones that may have formed are removed.

## Discussion and conclusion

Long- term urethral catheters in neurologically impaired female patients result in dilatation of the urethra and severe incontinence. Patients presenting urine leakage are usually treated with a larger catheter, anticholinergics and/or β_3_ adrenoceptor agonists. Conventional management of this condition has been urethral closure (abdominal or vaginal) and the simultaneous placement of a suprapubic catheter. Postoperative detrusor spasms represent a significant concern because they could probably increase the rate of fistula formation and postoperative incontinence. During the initial postoperative period, it is critical to continue the treatment with anticholinergics and/or β_3_ adrenoceptor agonists [10, 11]. The transvaginal closure of urethra and anterior colporrhaphy ensure low morbidity and high success rates. The complete mobilization of bladder opening prevents high rates of a postoperative urinary fistula. At long-term follow-up of this procedure, 50% of the women were dry after the initial operation. Other complications encountered were bladder stones (21%), leakage around the catheter (17%), recurrent catheter blockage (10%), and urinary tract infections [11].

The functional urethral closure with pubovaginal sling [12], tension-free vaginal tape (TVT) [13], or a modified endoscopic colposuspension where sutures are tied more closely than for patients with genuine stress incontinence [14], represent alternative proposals. Careful patient selection for the aforementioned surgical methods is necessary because females with a completely eroded bladder neck and proximal urethra are not candidates for this procedure. They represent pilot studies with small number of patients and success rates up to 70%.

Because patients with advanced neurological conditions are at high surgical risk, the transvaginal approach may be the ideal option. It provides a good exposure, excellent tolerance and low morbidity. It can perform in obese patients and the very unfit disabled patients even under local anaesthetic. However, there are very few reports about the optimal treatment of a large bladder opening in female patients who are neurologically impaired.

For patients who do not desire or are unable to perform regular intermittent catheterization, the options include bladder neck closure done in conjunction with ileovesicostomy or construction of an ileal conduit. The main reason for choosing a conduit over ileovesicostomy is determined by the stoma site. Patients who use a wheelchair benefit from a stoma in a higher position on the abdominal wall and this may be difficult to reach for an ileovesicostomy [15].

Umbilical location is an ideal position for cosmetic reasons, especially for patients willing to perform CISC. Mitrofanoff appendicovesicostomy represents a convenient way to construct a continent abdominal, urinary stoma [16]. The advantages of appendix diversion include maintaining complete continence, easy catheterization; excellent body image; and rarity of post-surgical complications such as dermatitis and urinary tract infection. Regarding the length of appendix, the cutaneous stoma can be placed in the umbilicus or the lower right abdominal quadrant [17, 18]. The submucosal tunnel and abdominal wall muscles are critical factors concerning the success rate of continence according to Yang et al. [19].

Continent catheterizable stomas display high rates of complications, including stomal stenosis requiring revision or conversion to an alternative channel (12–30% of cases). Usually, an ileal conduit is the alternative stoma, but a stomal appliance is a significant drawback for resource-deprived families [20].

We performed an anterior colporrhaphy to ensure the transposition of the bladder gap to a new position, high behind the symphysis pubis. The multiple layers and the special care for the underlying layers without apposition of the suture lines diminish the rates of fistula. We used the Mitrofanoff technique, usually presented in children, to create a continent catheterizable stoma as an adjunct to continent urinary tract reconstruction.

This case demonstrates that in the era of CISC, there are still neurologically impaired females suffering from rare but critical adverse effects of indwelling catheters. The urethra and bladder neck erosion represent a demanding treatment assignment. The combined use of two surgical approaches (Mitrofanoff technique for continent stoma and transvaginal closure of urethra with anterior colporrhaphy) represent rare but lifesaving potential treatment.

## Data Availability

Data are available from the corresponding author on reasonable request.

## Abbreviations

CISC: Clean intermittent self-catheterization
CT: Computed tomography
TVT: Tension-free vaginal tape
