# Correction to: Transvaginal closure of urinary bladder opening and Mitrofanof technique in a neurologically impaired female with chronic indwelling catheter: a case presentation

**DOI:** 10.1186/s12894-022-00981-1

**Published:** 2022-03-16

**Authors:** Athanasios Zachariou, Minas Paschopoulos, Aris Kaltsas, Fotios Dimitriadis, Athanasios Zikopoulos, Charalampos Mamoulakis, Atsushi Takenaka, Nikolaos Sofkitis

**Affiliations:** 1 Urology Department, Medical School, University of Ioannina, Ioannina, Greece; 2 Department of Obstetrics and Gynaecology, Medical School, University of Ioannina, Ioannina, Greece; 3 Urology Department, Medical School, Aristotle University of Thessaloniki, Thessaloniki, Greece; 4 Urology Department, Medical School, University of Crete, Heraklion, Greece; 5 Urology Department, Medical School, Tottori University, Yonago, Japan; 6 3 Spyridi Street, 38221 14 Volos, Greece

## Correction to: Zachariou et al. BMC Urol (2021) 21:93 10.1186/s12894-021-00861-0

Following publication of the original article [1], the authors identified that the given name and family name were erroneously transposed.

The incorrect authors name is:

Zachariou Athanasios, Paschopoulos Minas, Kaltsas Aris, Dimitriadis Fotios, Zikopoulos Athanasios, Mamoulakis Charalampos, Takenaka Atsushi and Sofkitis Nikolaos

The correct authors name is:

Athanasios Zachariou, Minas Paschopoulos, Aris Kaltsas, Fotios Dimitriadis, Athanasios Zikopoulos, Charalampos Mamoulakis, Atsushi Takenaka and Nikolaos Sofkitis

The author group has been updated above and the original article [1] has been corrected.

